# Role of *ASXL1* and *TP53* mutations in the molecular classification and prognosis of acute myeloid leukemias with myelodysplasia-related changes

**DOI:** 10.18632/oncotarget.3460

**Published:** 2015-02-28

**Authors:** Raynier Devillier, Véronique Mansat-De Mas, Veronique Gelsi-Boyer, Cecile Demur, Anne Murati, Jill Corre, Thomas Prebet, Sarah Bertoli, Mandy Brecqueville, Christine Arnoulet, Christian Recher, Norbert Vey, Marie-Joelle Mozziconacci, Eric Delabesse, Daniel Birnbaum

**Affiliations:** ^1^ Hematology Department, Institut Paoli Calmettes, Marseille, France; ^2^ Aix-Marseille University, Marseille, France; ^3^ Département d'Oncologie Moléculaire, Centre de Recherche en Cancérologie de Marseille (CRCM), Institut Paoli-Calmettes, UMR1068 Inserm, Marseille, France; ^4^ Biopathology Department, Institut Paoli Calmettes, Marseille, France; ^5^ Hematology Department, Institut Universitaire du Cancer Toulouse–Oncopole, Toulouse, France; ^6^ Toulouse University, Toulouse, France

**Keywords:** acute myeloid leukemia, myelodysplasia-related changes, mutational status, ASXL1, TP53

## Abstract

Acute myeloid leukemias (AML) with myelodysplasia-related changes (AML-MRC) are defined by the presence of multilineage dysplasia (MLD), and/or myelodysplastic syndrome (MDS)-related cytogenetics, and/or previous MDS. The goal of this study was to identify distinct biological and prognostic subgroups based on mutations of *ASXL1*, *RUNX1*, *DNMT3A*, *NPM1*, *FLT3* and *TP53* in 125 AML-MRC patients according to the presence of MLD, cytogenetics and outcome. *ASXL1* mutations (n=26, 21%) were associated with a higher proportion of marrow dysgranulopoiesis (mutant vs. wild-type: 75% vs. 55%, p=0.030) and were mostly found in intermediate cytogenetic AML (23/26) in which they predicted inferior 2-year overall survival (OS, mutant vs. wild-type: 14% vs. 37%, p=0.030). *TP53* mutations (n=28, 22%) were mostly found in complex karyotype AML (26/28) and predicted poor outcome within unfavorable cytogenetic risk AML (mutant vs. wild-type: 9% vs. 40%, p=0.040). In multivariate analysis, the presence of either *ASXL1* or *TP53* mutation was the only independent factor associated with shorter OS (HR, 95%CI: 2.53, 1.40-4.60, p=0.002) while MLD, MDS-related cytogenetics and previous MDS history did not influence OS. We conclude that *ASXL1* and *TP53* mutations identify two molecular subgroups among AML-MRCs, with specific poor prognosis. This could be useful for future diagnostic and prognostic classifications.

## INTRODUCTION

In the WHO 2008 classification, acute myeloid leukemia (AML) with myelodysplasia-related changes (AML-MRC) is defined as a distinct entity by the presence of multilineage dysplasia (MLD), and/or myelodysplastic syndrome (MDS)-related cytogenetics, and/or previously diagnosed MDS or MDS/Myeloproliferative neoplasm (MDS/MPN) [1]. The prognostic value of these three criteria is not established. The independent prognostic value of MLD is controversial and varies among different subsets of AML [2-7]. AML with MDS-related cytogenetics or previously diagnosed MDS or MDS/MPN have often unfavorable cytogenetics, and are associated with poorer outcome than AML without criteria of AML-MRC [2, 8, 9].

Although gene mutations are now a major tool for AML classification into distinct entities with specific prognosis [10-12], no molecular pattern is currently associated with AML-MRC. We hypothesized that the presence of mutations in targeted genes of interest could help identify subgroups of AML-MRC with distinct biological features and specific outcome. We had previously reported that AML-MRC have a specific mutation pattern sharing mutations found in both AML and high risk MDS and a particularly high frequency of *ASXL1* mutation [13]. We report here a cohort of patients with AML-MRC for whom we analyzed the presence of mutational events according to AML-MRC criteria (MLD, cytogenetics and patient history) and identified mutation-based subgroups with specific poor outcome.

## RESULTS

### Patient, disease and treatment characteristics

We studied 149 patients. After morphological review, 24 patients were excluded from the main analysis because of not enough dysplasia to reach the MLD criteria. These patients were analyzed separately (Supplemental Table 1). The remaining 125 fitting the AML-MRC criteria were considered for the main analysis and their characteristics are reported in Table 1. Median age was 71 years (range: 18-90). Fifty-nine patients (47%) had a previously diagnosed MDS. Seventy-one patients (57%) had MDS-related cytogenetics, including 42 patients (34%) with complex karyotype AML (CK-AML). Ninety-four patients were evaluable for morphological dysplasia by the double centralized review. Multilineage dysplasia (MLD) was found in 38/94 patients (40%). Sixty-seven patients (54%) received intensive induction chemotherapy. Mutations were found in *ASXL1* (n = 26, 21%), *RUNX1* (n = 15, 12%), *DNMT3A* (n = 11, 9%), *NPM1* (n = 4, 3%), *FLT3* (n = 9, 7%) and *TP53* (n = 28, 22%). No mutation was found in 47 (38%) patients (31 with non-complex karyotype NCK-AML and 16 with CK-AML).

**Table 1:** Characteristics of the 125 patients with criteria for AML-MRC

	All patients	(N = 125)
	N	%
**Median age (years, [range])**	71	[18-90]
**FAB Classification**		
0	6	5%
1	13	10%
2	44	35%
4	25	20%
5	9	7%
6	9	7%
Unclassifiable	19	15%
**Previously diagnosed MDS**	59	47%
Multilineage dysplasia*	38	40%
**Cytogenetics**		
Normal	28	22%
Abnormal non-complex	55	44%
Complex	42	34%
Monosomal	36	29%
Non monosomal	6	5%
**MDS-related cytogenetics**	71	57%
**Cytogenetic risk group**		
Intermediate	65	52%
Unfavorable	60	48%
**Treatment**		
Intensive chemotherapy	67	54%
Non-intensive chemotherapy	31	25%
*Demethylating agent*	*19*	15%
*Other*	*12*	10%
Supportive care	27	22%

* Multilineage dysplasia was evaluated on 94 patients MDS = myelodysplastic syndrome

### Mutation profiles according to previous history of MDS, cytogenetics and MLD

**Figure 1:**
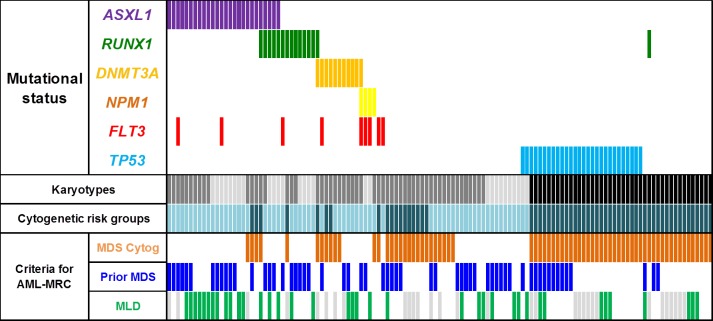
Co mutation profile of *ASXL1, RUNX1, DNMT3A, NPM1, FLT3-ITD* and *TP53* genes in the 125 patients with AML-MRC: Karyotypes: normal (light grey), abnormal non-complex (dark grey) and complex (black) Cytogenetics risk group: intermediate (light blue) and unfavorable (dark blue). MLD: green bars indicate the presence of criteria for MLD and grey bars are samples that were not evaluable for MLD. These latter had at least one other criteria for AML-MRC to enrolled them in this study. MDS = myelodysplastic syndrome; MLD = multilineage dysplasia.

*TP53* mutations were exclusive from all other mutations and all but two (26/28, 93%) were found in CK-AML. Mutations in the other genes were almost exclusively found in NCK-AMLs (Table 2A, Figure 1). In NCK-AMLs, *ASXL1* was the most frequently mutated gene (26/83, 31%). These mutations were associated with the absence of MDS-related cytogenetics (MDS-related cytogenetics: yes vs. no: 4/29, 14% vs. 22/54, 41%, p = 0.013). Other mutations were equally distributed whether MDS-related cytogenetics was present or not (Figure 1).

**Table 2:** Frequencies of mutation in *ASXL1*, *RUNX1*, *DNMT3A*, *NPM1*, *FLT3-ITD* and *TP53* in the 125 AML-MRC patients and according to the karyotypes (A) and to the cytogenetic risk groups(B)

A	NCK-AML	(N=83)	CK-AML	(N=42)	p
	N	%	N	%	
***ASXL1***	26	31%	0	0%	<0.001
***RUNX1***	14	17%	1	2%	0.004
***DNMT3A***	11	13%	0	0%	<0.001
***NPM1***	4	5%	0	0%	0.243
***FLT3-ITD***	9	11%	0	0%	0.059
***TP53***	2	2%	26	62%	<0.001
CK = complex karyotype; NCK = Non complex karyotype					

B	Intermediate (N=65)		Unfavourable (N=60)		p
	N	%	N	%	
***ASXL1***	23	35%	3	5%	<0.001
***RUNX1***	11	17%	4	7%	0.067
***DNMT3A***	8	12%	3	5%	0.130
***NPM1***	4	6%	0	0%	0.070
***FLT3-ITD***	8	12%	1	2%	0.022
***TP53***	2	3%	26	43%	<0.001

We analyzed the presence of mutations according to the cytogenetic risk group (Table 2B). In the 94 patients evaluable for MLD, patients with criteria for MLD had more *ASXL1* mutations (MLD: 15/38, 39% vs. no MLD: 8/48, 14%, p = 0.007). We did not find any correlation between the presence of MLD and other mutations. Median percentages of bone marrow DGP, DEP and DMP in the 94 patients were 64%, 24% and 45% respectively. *ASXL1* mutations were associated with higher DGP (*ASXL1-mut*: 75% vs. *ASXL1-wt*: 55%, p = 0.030) but similar DEP (*ASXL1-mut*: 20% vs. *ASXL1-wt*: 25%, p = 0.933) and DMP (*ASXL1-mut*: 53% vs. *ASXL1-wt*: 40%, p = 0.139). There was no difference in percent of morphologic dysplasia according to any other genes (Supplemental Table 2). In a linear regression analysis including the mutation status of the 6 genes, mutation of *ASXL1* remained associated with higher DGP (Coefficient beta = 20, p = 0.023, Supplemental Table 3). The frequencies of these mutations were not different according to the presence or not of prior MDS or MDS/MPN.

### Outcome after intensive chemotherapy in AML-MRC patients

Among the 125 AML-MRC patients, only the 67 patients who received intensive induction chemotherapy were considered for the outcome analyses. Among them, 42 achieved CR (63%). Cytogenetic risk group, the presence of MDS-related cytogenetics, previous history of MDS or MDS/MPN, and MLD did not influence the CR rate (data not shown). The presence of an *ASXL1* mutation was associated with a lower CR rate (*ASXL1*-mut vs. *ASXL1*-wt: 40% vs. 69%, p = 0.039). Other gene mutations did not influence the CR rate.

**Figure 2:**
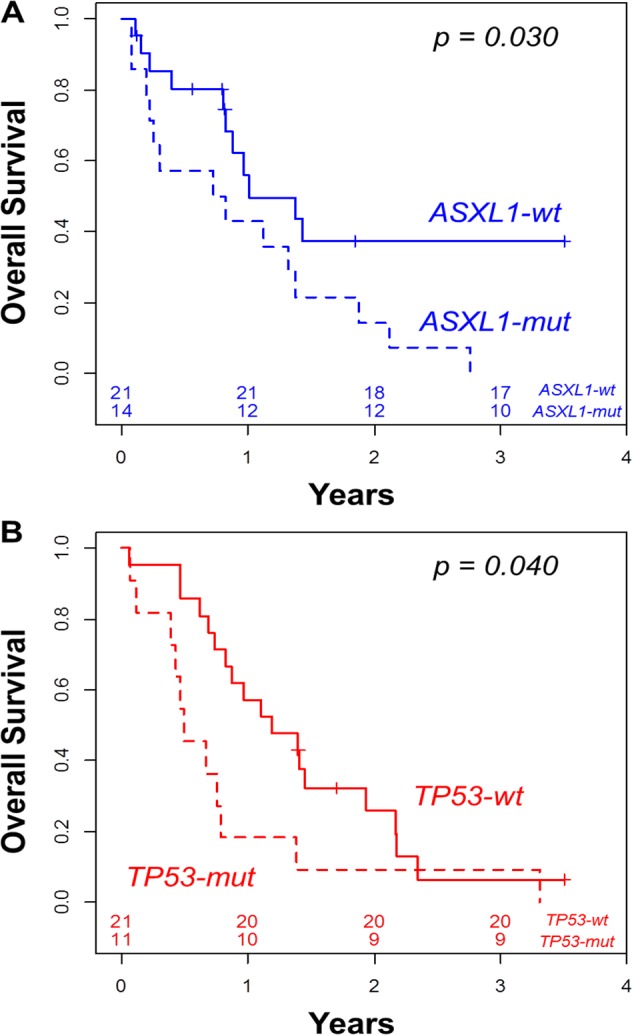
Overall survival according to the presence *ASXL1* mutation in the intermediate cytogenetic patients (A) and according to the presence of *TP53* mutation in the unfavorable cytogenetic patients (B)

The 2-year OS was 24% in the 67 intensively treated patients. Cytogenetics did not predict outcome, with a 2-year OS of 27% and 20% in the intermediate and unfavorable groups, respectively (p=0.351, Table 3). Similarly, MDS-related cytogenetics, previous history of MDS or MDS/MPN, and MLD did not significantly influence OS (data not shown). Among the intermediate cytogenetic (IC-AML) patients, the presence of an *ASXL1* mutation was associated with worse 2-year OS (14%) compared to patients without *ASXL1* mutation (37%, p = 0.030, Figure 2A, Table 3).

**Table 3:** Univariate analyses of overall survival

	N	2-year OS	(95%CI)	p
**All patients**	67	24%	(15-37)	
**Cytogenetics**				
Intermediate	35	27%	(15-49)	0.351
Unfavorable	32	20%	(10-41)	
**Intermediate risk**				
*ASXL1-wt*	21	37%	(20-70)	0.030
*ASXL1-mut*	14	14%	(4-52)	
**Unfavorable risk**				
*TP53-wt*	21	26%	(12-56)	0.040
*TP53-mut*	11	9%	(1-59)	
**Genotype**				
*No mutation*	41	29%	(17-49)	0.005
*ASXL1 or TP53 mut*	26	15%	(7-38)	

In the unfavorable cytogenetic (UC-AML) group, *TP53*-mutated patients had a lower 2-year OS (9%) than *TP53-wild type* patients (26%, p = 0.040, Figure 2B, Table 3). In multivariate analyses adjusted for age and WBC, IC-AML with mutated *ASXL1* (HR = 2.67, 95%CI = [1.15-6.24], p = 0.023) and UC-AML with mutated *TP53* (HR = 5.44, 95%CI = [2.16-13.65], p < 0.001) had shorter OS than IC-AML with wild type *ASXL1* (considered as reference, HR = 1). Of note, patients with UC-AML with wild type *TP53* (HR = 1.14, 95%CI = [0.52-2.50], p = 0.743) had similar OS compared to those with intermediate cytogenetic and no *ASXL1* mutation (considered as reference, HR = 1).

**Figure 3:**
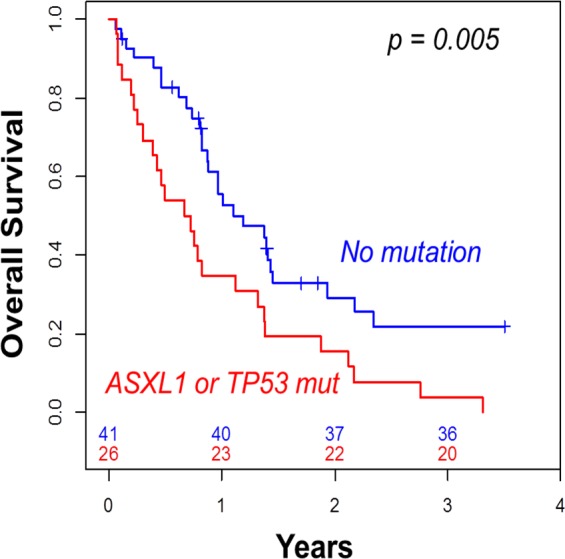
Overall survival according to the presence of *ASXL1* or *TP53* mutation

Using only the mutational status of *ASXL1* and *TP53* to stratify patient outcome, we found that patients who presented with either *ASXL1* or *TP53* mutation had worse 2-year OS (15%) than those with both *ASXL1* and *TP53* wild type (29%, p = 0.005, Table 3, Figure 3). In multivariate analysis including age, WBC and cytogenetics, the presence of either *ASXL1* or *TP53* mutation remained the only independent predictive factor associated with both lower CR rate (HR = 0.29, 95%CI = [0.09-0.89], p = 0.031) and shorter OS (HR = 2.53, 95%CI = [1.40-4.60], p = 0.002).

### Patients with dysplasia but without criteria for MLD

These 24 patients were separately analyzed because they did not reach AML-MRC criteria after morphological review. Indeed, they had no previous history of MDS or MDS/MPN and no MDS-related cytogenetics. Morphological review showed dysplasia that did not reach criteria for MLD. All of them had intermediate cytogenetics and 15 (63%) had normal karyotype (Supplemental Table 1). Among these 24 patients, median percentage of DGP, DEP and DMP was 50% (range: 7-97%), 20% (range: 0-45%) et 26% (range: 0-83%), respectively. We found 4 mutations in *ASXL1* (17%), 5 in *RUNX1* (21%), 1 in *DNMT3A* (4%), 2 in *NPM1* (8%), 4 in *FLT3* (17%) and no *TP53* mutation. *ASXL1* mutated patients had a median percent of DGP of 62% (range: 35-90%) vs. 50% (range: 7-97%) in those with wild type *ASXL1.*

## DISCUSSION

We previously reported that AMLs with MRC harbor a specific mutational profile with a high proportion of *ASXL1* and *RUNX1* mutations and less *DNMT3A*, *FLT3* and *NPM1* mutations than AMLs without criteria of AML-MRC [13]. In this series including only patients with AML-MRC, we have evaluated the correlation between these mutations and the different criteria defining AML-MRC (previous history of MDS or MDS/MPN, MDS-related cytogenetics, and MLD). We found that none of the three MRC criteria could help identify a specific mutational profile within AML-MRC, suggesting that these criteria do not have a molecular basis, at least within the limit of the genes studied. This heterogeneity in terms of biological features as well as in prognostic significance suggests that AML-MRC is not a true distinct entity. Although they are defined as AML-MRC because of MDS-related cytogenetics, CK-AML should be considered separately because of the presence of a specific mutational profile consisting in a high frequency of *TP53* mutations and the absence of other mutations. As expected, *TP53* mutations predicted a worse outcome among patients with unfavorable cytogenetics, supporting that genetic stratification is useful to predict differential patient outcome although classified in the same cytogenetic risk group. This is in line with previous reports showing the close correlation between *TP53* mutation, CK-AML and poor outcome [18, 19]. In NCK-AML, we found that *ASXL1* was the most frequently mutated gene (31%). It was the only mutation associated with the presence of MLD and a higher proportion of DGP in bone marrow. This is in agreement with previous biological reports showing the role of *ASXL1* in the appearance of dysplasia and myeloid transformation [20-23]. However, the overlap between *ASXL1* mutations and morphological MLD was not complete, because 39% of patients with MLD had *ASXL1* mutation and 14% patients without MLD had *ASXL1* mutation. Thus, there is no strong association between morphological analyses and the presence of an *ASXL1* mutation. Interestingly, *ASXL1* mutations were associated with the absence of MDS-related cytogenetics among the NCK-AML, as if *ASXL1* mutations and karyotype with MDS-related abnormalities were mutually exclusive. Finally, there was no correlation between *ASXL1* mutation and previous history of MDS or MDS/MPN.

Taken together, these results suggest that the presence of an *ASXL1* mutation could be considered as an independent molecular marker of dysplasia in AML that is not redundant with the criteria defining AML-MRC. This could be helpful in the perspective of developing a molecular classification of AML-MRC. This statement is also supported by the presence of a specific gene expression profile associated with *ASXL1*-mutated AML [24]. Like *TP53* stratification of outcome in UC-AML, *ASXL1* mutations were associated with a shorter OS in intermediate risk patients, with a 2-year OS (14%) close to that observed in unfavorable cytogenetic patients (20%). Different studies that did not specifically focus on AML-MRC also reported this poor outcome associated to *ASXL1* mutations [25, 26]. Thus, stratification upon *ASXL1* and *TP53* mutation, which identified two distinct biological subgroups among AML-MRC, was the only significant predictor of outcome while usual cytogenetic risk classification or the different criteria defining AML-MRC (prior MDS or MDS/MPN, MDS-related cytogenetics and MLD) failed to predict patient outcome in our series. Because of the limited number of patients in some subgroups, these results need to be confirmed in larger studies.

Although *ASXL1* and *TP53* mutations could classify distinct subgroups of AML-MRC, patients without any of these mutations still represent a heterogeneous group of AML-MRC harboring different morphological, cytogenetic and molecular features. The mutations of *DNMT3A* and/or *NPM1*, which are usually found in *de novo* AML and are mutually exclusive with *ASXL1* and *TP53* mutations, could identify patients for whom the definition of AML-MRC should be questioned. Falini et al. reported that MLD did not identify distinct biological and clinical entities among *NPM1*-mutated AML, with overlapping gene expression profiling and similar outcome [4]. In contrast, some patients had morphological dysplasia but not enough to reach MLD criteria, leading to their exclusion from the AML-MRC category in the absence of other criteria. Among these patients, those who presented with *ASXL1* mutations might be included in the same subgroup as *ASXL1*-mutated AML-MRC. This supports the need of redefining AML-MRC upon molecular abnormalities.

Finally, we did not find any mutation in the 6 genes studied in 47 patients, suggesting the need to identify other molecular markers to stratify these patients. Because *ASXL1* is a key regulator of the polycomb repressive complex 2 (PRC2), it is possible that AML carrying alterations of other genes involved in methylation marks via PRC2 and myeloid transformation such as *EZH2* [27, 28], *JARID2* [29] or *BAP1* [30, 31] could share characteristics of *ASXL1*-mutated AML. This could help identify AML-MRC through a common altered molecular pathway and could help develop future targeted treatments. An extensive mutational screening using whole genome or exome sequencing could be useful in this setting to improve the molecular characterization of these unmutated cases.

We conclude that the criteria defining AML-MRC do not identify distinct clinical and biological subgroups and do not predict outcome of patients with AML-MRC. In contrast, *ASXL1* and *TP53*-mutated AML identify two distinct biological subgroups of AML-MRC with very poor outcome. This molecular characterization could be useful to redefine AML-MRC in a future classification aiming at merging biological characterization and specific prognostic value.

## PATIENTS AND METHODS

### Selection criteria

We conducted a retrospective analysis of patients from two French centers. Selection criteria for analyses were: (i) Diagnosis of AML with criteria for AML-MRC according to the WHO classification[1]: previously diagnosed MDS or MPN/MDS; and/or multilineage dysplasia (MLD); and/or MDS-related cytogenetic abnormalities (complex karyotype (CK) defined by three or more chromosomal abnormalities, -7 or del(7q); -5 or del(5q); i(17q) or t(17p); -13 or del(13q); del(11q); del(12p) or t(12p); del(9q); idic(X)(q13); t(11;16)(q23;p13.3); t(3;21)(q26.2;q22.1); t(1;3)(p36.3;q21.1); t(2;11)(p21;q23); t(5;12)(q33;p12); t(5;7)(q33;q11.2); t(5;17)(q33;p13); t(5; 10)(q33;q21); t(3;5)(q25;q34); (ii) Genomic DNA available for mutational analyses.

AML with inv(3)/t(3;3), t(6;9) and t(v;11q23) or with favorable risk according to the ELN classification (inv(16)/t(16;16), t(8;21), t(15;17), normal karyotype AML with NPM1 or CEBPA mutations) were excluded as well as therapy-related AML [14].

### Morphological analyses

Smears of bone marrow aspirates made at diagnosis were collected and stained by May-Grünwald-Giemsa. Each smear was retrospectively analyzed by two expert cytologists in both centers. We assessed the percentage of dysplastic cells in each lineage. MLD was assessed according to the WHO classification criteria: at least 50% of dysplastic cells in at least 2 lineages. We established the percentage of dysgranulopoiesis (DGP), dyserythropoiesis (DEP) and dysmegakaryopoiesis (DMP) for each smear.

### Mutational analyses

Direct sequencing was done using the Sanger method as previously described [15]. We searched for mutation of *ASXL1* (exon 12), *RUNX1* (exon 1-8), *DNMT3A* (exon 15-23), *NPM1* (exon 12), *FLT3* (internal tandem duplication ITD, exon 14-15) and *TP53* (exon 4-10).

### Statistical analyses

Chi-square or Fischer tests were used to compare categorical variables. We used non-parametric test (U Mann Whitney) to compare the median percentage of dysplastic cells in each lineage according to mutational status for each gene. As multivariate model, linear regression was used to find correlation between morphologic dysplasia and the presence of mutation. Survivals were calculated using the Kaplan Meier estimator [16]. Time to event started from the date of diagnosis. We compared survivals using the Log-Rank test. Cox regression was used for multivariate of OS [17]. Statistics were computed using the R.3.1.0 software.

## SUPPLEMENTARY MATERIALS AND TABLES


