# Supplementary material for: Role of ASXL1 and TP53 mutations in the molecular classification and prognosis of acute myeloid leukemias with myelodysplasia-related changes
Source: Oncotarget. 2015 Feb 28;6(10):8388–96. doi: 10.18632/oncotarget.3460 (PMC4480760; doi:10.18632/oncotarget.3460)
Supplement: Supplementary file 1 [file oncotarget-06-8388-s001.pdf]

Role of *ASXL1* and *TP53* mutations in the molecular classification and prognosis of acute myeloid leukemias with myelodysplasia-related changes

Supplementary Material

Supplemental Table 1: Characteristics (A) and mutation profile (B) of the 24 patients with dysplasia not reaching criteria for MLD

A) Characteristics

Patients with dysplasia without WHO criteria for MLD (N = 24)	N	%
**Age, Median (range)**	68	(45-68)
**WBC, Median (range)**	3.7	(0.7-140)
**FAB Classification**		
1	2	8%
2	10	42%
4	6	25%
5	1	4%
6	3	13%
Unclassifiable	2	8%
**Cytogenetics**		
Normal	15	63%
Trisomy 8	4	17%
Trisomy 21	2	8%
Trisomy 12	1	4%
inv(9)	1	4%
t(2;9)	1	4%

MLD = multilineage dysplasia, WBC = white blood cells

B) Mutation profile

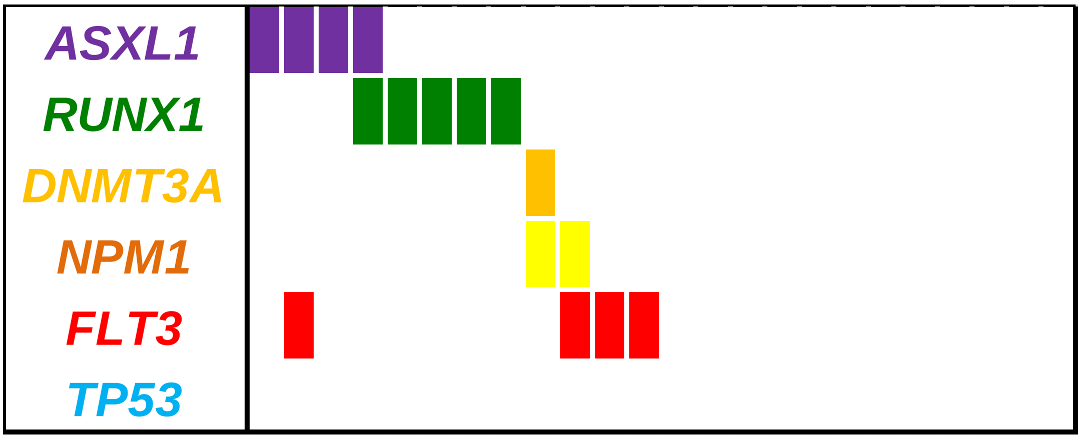

Supplemental Table 2: Median proportion of dysplastic cells in each lineage according to the presence of mutations

	Percent DGP		p	Percent DEP		p	Percent DMP		p
	N	Median		N	Median		N	Median	
**All patients**	90	64%	-	84	24%	-	90	45%	-
ASXL1									
wt (n=71)	**67**	**55%**	**0.030**	64	25%	0.933	70	40%	0.139
mut (n=23)	**23**	**75%**		20	20%		20	53%	
RUNX1									
wt (n=83)	79	65%	0.478	74	23%	0.819	80	45%	0.299
mut (n=11)	11	55%		10	28%		10	30%	
DNMT3A									
wt (n=87)	83	63%	0.389	79	25%	0.842	83	44%	0.916
mut (n=7)	7	75%		5	20%		7	45%	
NPM1									
wt (n=90)	87	64%	0.232	83	24%	0.086	86	45%	1.000
mut (n=4)	3	13%		1	80%		4	43%	
FLT3									
wt (n=87)	84	65%	0.441	79	24%	0.609	83	45%	0.062
ITD (n=7)	6	43%		5	30%		7	0%	
TP53									
wt (n=74)	70	64%	0.880	64	20%	0.112	70	45%	0.271
mut (n=20)	20	60%		20	30%		20	32%	

DEP = dyserythropoiesis; DGP = dysgranulopoiesis; DMP = dysmegakaryopoiesis; mut = mutant; wt = wild type; ITD = internal tandem duplication

Supplemental Table 3: Linear regression for proportion of dysgranulopoeisis according to the presence of gene mutations

	**Coefficient beta**	**Standard deviation**	**p**
ASXL1	20.389	8.824	0.023
RUNX1	-8.026	11.148	0.474
DNMT3A	17.904	13.879	0.201
NPM1	-26.058	22.304	0.246
FLT3	-0.846	15.894	0.958
TP53	2.346	9.405	0.804
